# The potential of water markets to allocate water between industry, agriculture, and public water utilities as an adaptation mechanism to climate change

**DOI:** 10.1007/s11027-015-9662-z

**Published:** 2015-06-11

**Authors:** Jason F. L. Koopman, Onno Kuik, Richard S. J. Tol, Roy Brouwer

**Affiliations:** 1 Department of Environmental Economics, Institute for Environmental Studies, VU University Amsterdam, De Boelelaan 1087, 1081HV Amsterdam, The Netherlands; 2 Department of Economics, University of Sussex, Sussex, UK; 3 Department of Spatial Economics, VU University Amsterdam, De Boelelaan 1087, 1081HV Amsterdam, The Netherlands

**Keywords:** Climate change, Water scarcity, Water markets, Computable general equilibrium

## Abstract

One of the climate change scenarios that have been developed for the Netherlands predicts hotter and drier summers and a substantial drop in river discharge. This might lead to water scarcity with detrimental economic and environmental effects. Among the possible adaptation responses to climate change-induced water scarcity, the re-allocation of water resources among competing uses should also be considered. In this paper, we extend and apply a computable general equilibrium (CGE) model to assess the potential of water markets (water allocation according to its shadow price) to guide the allocation of scarce water across agriculture, manufacturing, and public water supply. We develop four scenarios in which the scope of water markets is increased from industry-specific to economy-wide. The results show that the agricultural sector bears nearly all of the losses from a new water-scarce climate, while the manufacturing sectors are able to mitigate their losses to a large extent by technical measures. Extending the scope of water markets unambiguously increases economic output and results in a re-allocation of water to the manufacturing sector from the agricultural sector and from public water services. If, perhaps for political reasons, public water services are excluded from water trading, water is re-allocated from agriculture to manufacturing. Depending on which sectors are included, the construction of a water market can have negative or positive effects on a sector’s output, and although the implementation of water markets may be positive for overall economic output and can hence assist adaptation, the effect on vulnerable or societally sensitive economic sectors, such as public water, should be taken into account when implementing such a market.

## Introduction

Global climate change exerts significant pressure on the way that we allocate our limited water resources across different water uses and user groups. Different countries apply different allocation rules, varying from national top-down command and control policies to local or regional water markets and transboundary river basin treaties. In designing adaptation responses to climate change, such as updating water infrastructure, economic analysis can play an important role in reducing costs and improving efficiency (Hughes et al. 2010).

Water allocation according to economic principles has been examined from various perspectives in the literature to explore very different types of problems. A few examples of these different perspectives are represented in Harou et al. (2009), who describe hydro-economic models which allocate water to maximize economic output within a river basin, Ansink and Ruijs (2008) who use a game theoretical model to examine the stability of water allocation agreements along a river, Rosegrant et al. (2002) who use a partial equilibrium model to forecast worldwide changes to agriculture, and Zhu and van Ierland (2012) who examine water allocation from a welfare perspective. Brouwer and Hofkes (2008) provide a discussion on the various perspectives of water modeling. There also exists a sizable literature that has addressed this issue from a computable general equilibrium (CGE) perspective, starting from Berck et al. (1990). Most of the studies which take a CGE perspective are on a national scale, for example, Letsoalo et al. (2007) for South Africa and Diao et al. (2005) for Morocco. A number of studies are on a world scale, for example Berrittella et al. (2007) and Calzadilla et al. (2010). Almost all CGE studies focus exclusively on water use in agriculture. A few notable exceptions are that of Goodman (2000) who uses a dynamic CGE to examine the effects of temporary transfers from rural to urban use in the Arkansas River basin as opposed to increased water storage and Gomez et al. (2004) who examine water rights exchanges between agriculture and urban manufacturing and tourism as an alternative to desalination plants in the Balearic islands. Both papers focus exclusively on a single region in their analysis. Berrittella et al. (2007) analyzed water use by industrial sectors as well as agricultural sectors at the global level. They used a fairly rigid formulation of water in production, however, which allowed for very little adaptation by economic agents and did not allow for competition for water between industry and agriculture. Ponce et al. (2012) recently reviewed this literature, and among their findings is the general lack of detail in non-agricultural sectors and in industrial water-using sectors in particular. Further, they found that most studies essentially examine a loss of water productivity instead of an explicit loss of water availability.

Our study builds on and adds to the existing literature by examining water scarcity in a general equilibrium framework across multiple economic sectors (agriculture, manufacturing, and public water services) in an international river basin context. We use a CGE model, which explicitly models raw water use in the agricultural, public water services, and manufacturing sectors to examine the economic impact of climate change-induced water scarcity on the Netherlands, and we compare various principles of water allocation as an adaptation response. We examine not only the effect of water allocation principles on the water-dependent sectors, but also the wider direct and indirect economic effects that result from water scarcity and the economy’s response to it. A particular innovation of our model is the inclusion of self-abstracted (raw) water in the non-agricultural sectors as separate from drinking water purchases, as well as an inclusion of an explicit description of the other countries that share the river basins of the rivers Rhine and Meuse with the Netherlands: Germany, Belgium, France, and Luxembourg. By placing the Dutch economy in such an international context, we can determine the wider economic impact of water scarcity and adaptation on the open Dutch economy.

Although the paper is about the Netherlands, its insights are wider. We contrast the impacts of climate change under alternative market configurations. We show that water markets have the potential to help reduce the negative impacts of climate change, as more options are opened for realigning water use in the face of increased scarcity. We show that although the consequences of implementing a water market may not always align with political goals such as high agricultural productivity or a low price for public water services, creating, extending, or improving water markets can assist adaptation policy if carefully implemented. We therefore also show that adaptation is about more than engineering works (e.g., irrigation) or behavioral change (e.g., shorter showers): Institutional change, here the creation of a water market, matters too. On a methodological note, most CGE analyses ignore water as a factor of production. Some CGEs include water for agriculture, but with a few exceptions, water use in other economic sectors is omitted. We show that other water use can be included in a CGE. We further show that including other water-using sectors in a water market with agriculture has a measurable effect on agricultural output.

## Methodological approach and data

### Water as an economic input

Freshwater is a natural resource that provides many economic and environmental services (Briscoe 2005). Young and Haveman (1985) already noted 30 years ago that water has unique physical properties, complex economic characteristics, and important cultural features that distinguish it from all other resources. The idea that water resources management can benefit from economic principles can also be defended (Briscoe 2005) but should always take account of other, non-economic values that may restrict the scope of these principles.

The main sources of freshwater are rivers, lakes, and groundwater deposits (Zhu and van Ierland 2012). In our simulations, we focus on surface water that is supplied by rivers and through precipitation. We assume that groundwater deposits are not (further) depleted, so groundwater plays no role in the analysis. In the Netherlands, large-scale abstraction of surface water is subject to a license, but there is no tariff per unit of abstraction, so no water price as such. If freshwater becomes scarce (meaning that not all demand can be satisfied at present conditions), water gets a shadow price. The shadow price of water that is used by a certain activity is the value added that would be created by increasing the supply of water by one unit. Without water markets, shadow prices will, as a rule, differ across different economic activities and different locations. For this study, the term water markets is defined rather loosely as a mechanism which allocates water for economic use according to its shadow price and accordingly equalizes the marginal shadow costs of water use across economic activities. We do not describe the institutional setup of such markets, nor the physical infrastructure and associated investment costs that may need to be in place for water markets to function properly, nor do we take transaction costs into account. In addition, water markets in our analysis should be considered more as a yearly market for water use rights (for example, in the form of an auction) rather than as a spot market to satisfy immediate short-term water use needs.

### The model

For our analysis, we use an extension of the well-known Global Trade Analysis Project (GTAP) model (Hertel 1999) called GTAP-Water (GTAP-W) (Calzadilla et al. 2010). GTAP is a comparative static CGE model of the global economy. GTAP-W extends the original GTAP model by adding an explicit treatment of irrigation water in crop agriculture and distinguishes between irrigated land and rainfed land.

The standard GTAP model (we use version 6) describes the interactions of decisions of consumers and producers in all markets. Consumers have preferences over private consumption goods, public goods, and savings, and they buy the consumption bundle that maximizes their utility, given their income, according to a constant difference elasticity (CDE) function. Producers maximize profits given a constant return to scale production technology for all firms. The competitive equilibrium in the model is characterized by clearance of all markets and by the zero-profit condition for all firms. The substitution between domestically produced and imported goods is imperfect, following the approach suggested by Armington (1969) to treat goods of different origin as different, non-homogeneous goods. The production function in GTAP is a nested constant elasticity substitution (CES) function. At the top level of the nest, intermediate inputs are combined with a value-added composite which consists of labor, capital, and land. The capital and labor endowments are perfectly mobile domestically, while land is imperfectly mobile. All endowments are immobile across regions.

**Fig. 1:**
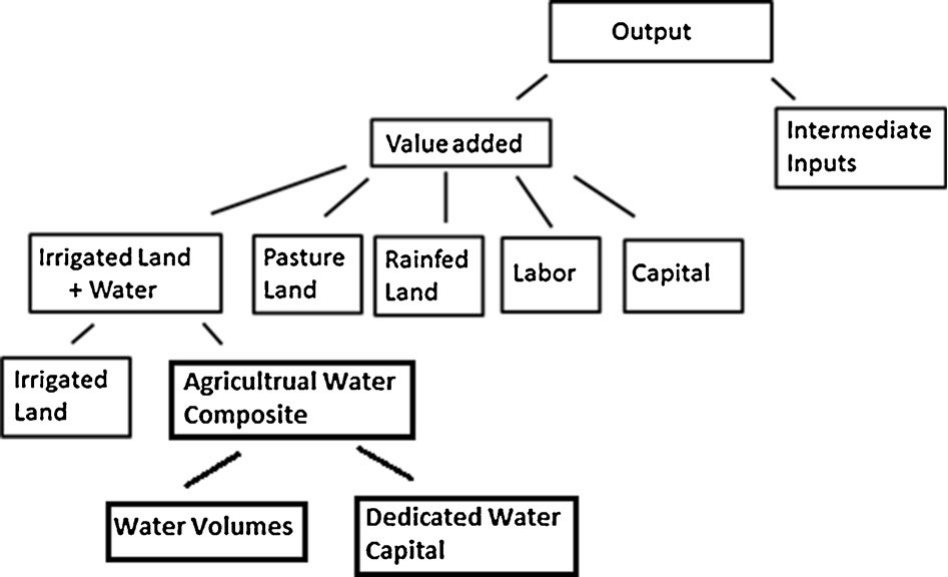
The constant elasticity of substitution (CES) production nest for the agricultural activities

GTAP-W extends the GTAP model by including more detail into the land endowment for agricultural producers (which are the exclusive users of the land endowment) splitting the original endowment into rainfed, irrigated, and pasture land and then further splitting off irrigation water from the irrigated land endowment. All of the new land and water endowments in GTAP-W inherit the partial mobility parameter from the original land endowment in GTAP. In the GTAP-W production function, in the lowest nest of the CES production function, crop farmers determine the level of irrigation based on the relative prices of land and irrigation water and the technical ease of varying the level of irrigation water on a given piece of land. In higher nests of the production function, the irrigated land is combined with capital, labor, and intermediate goods (seeds, fertilizers, pesticides) to produce an output like wheat or potatoes. Figure 1 provides a visual representation of the production function for the agricultural activities. The components of the agricultural water composite are discussed below.

We have extended the GTAP-W model in two ways.

**Fig. 2:**
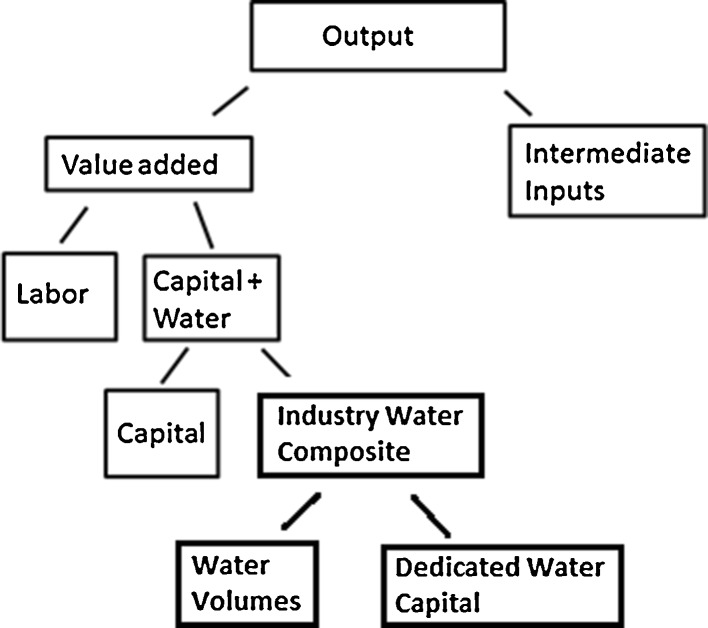
The constant elasticity of substitution (CES) production nest for non-agricultural activities

First, we extend the existing GTAP-W model by including freshwater use in the animal husbandry sector, manufacturing sectors, and the public water services sector (which delivers drinking water to households and firms). In the animal husbandry sector, the demand for water is modeled in the same way as the demand for irrigation water in crop agriculture: water is combined with pasture land on the basis of relative prices and technical possibilities. In contrast, the manufacturing and public water services sectors combine water with capital (not land). The assumption of the possibility of substitution between water and capital is in line with the findings of Dupont and Renzetti (2001) and Renzetti (1992) who assert that intake water may be a substitute for recirculation water which is more capital intensive. The assumption is also in line with Gomez et al. (2004) and Goodman (2000) who combine water with capital in their models in a similar way. Solely for the purpose of the policy simulations that are carried out later in this paper, we distinguish between water from the agricultural water market that is combined with land in the agricultural sectors and water from the industry water market that is combined with capital in the manufacturing and public water services sector. Note that we also use the terms agricultural water market and industry water market rather loosely as described in Sect. 2.1. In the case when the sectoral water endowments are fixed and non-tradable, we use the terms to describe the aggregate sectoral water endowments used separately in the agricultural and industry sectors. Figure 2 provides a visual representation of the production function for the non-agricultural activities, and the components of the industry water composite are described below.

Second, we extended the model by accounting for *volume* flows of water between sectors. This addition is necessary to insure a physical water balance when water is exchanged between very different types of use. In GTAP-W as presented in Calzadilla et al. (2010), the agricultural water endowment (renamed here the agricultural water composite) represents all of the benefits (and also the expenditure) of irrigation for production (for detail see Sect. 2.3 below). This includes everything involved in irrigation, not only physical water but also irrigation equipment. We make the same assumption that the values of the agricultural and industrial water composite include not only the water itself but also the value of all of the necessary machinery for the water activity.

As the agricultural water endowment in GTAP-W from Calzadilla et al. (2010) is measured in millions of dollars, any redistribution of the endowment among agricultural sectors redistributes the value of irrigation without an explicit accounting of water volumes. If one assumes that the added value of a cubic meter of water is roughly the same for all agricultural sectors, then this is a reasonable structure for examining a market for water (or for water use rights) in agriculture. However, our paper examines water redistribution where the ratio between the value of the water activity in production (the value of the agriculture or industry water composite) and the volume of water involved in that activity can vary quite substantially between uses, and this needs to be accounted for to insure that the volume of water before reallocation is the same as the volume after reallocation (see Sect. 2.3, specifically Table 3 for the magnitude of this difference for the Netherlands).

To separate the value of physical water from the rest of the agricultural water endowment, we have changed the name of agricultural water ‘endowment’ to agricultural water ‘composite’, and split up it further into physical water volumes and dedicated agricultural water capital (see Fig. 1). Similarly, for the public water services sector and the manufacturing sectors, we first split off the industry water composite from the rest of the capital endowment where the industry water composite includes the value of all expenditures on water-related activities of abstraction, purification, use in production, and discharge. We then further split the industry water composite into the value of physical water volumes and dedicated industry water capital, which represents the value of water equipment (see Fig. 2). Dedicated water capital is immobile. Physical water volumes are mobile in principle, but the mobility is restricted in various policy scenarios to simulate the various water market alternatives. The physical water volume endowment is combined with dedicated water capital with Leontief production technology (no substitution allowed). The immobility of dedicated water capital and the Leontief production technology between dedicated water capital and physical water volumes means that the value of the water composite is completely determined by the amount of physical water available and that a percentage reduction in amount of available water for production results in the same percentage reduction in the water composite. The elasticity of substitution between the irrigated land endowment and the agricultural water composite is 0.05. The elasticity of substitution between capital and the industrial water composite for the manufacturing sectors is 0.5 and is 0.1 for public water services. The elasticities of substitution were calibrated such that a 10 % reduction in water availability would induce the same price water elasticities that are given in Rosegrant et al. (2002).

A sensitivity analysis of the results with respect to the new elasticities introduced into the model, of substitution between land and water for agriculture and between capital and water for manufacturing and public water services, has been performed. The main conclusions on the economy-wide impact of water and on the direction of the movement of water in response to the water market policy scenarios (see Table 5, Sect. 4) are insensitive to the specific values of the substitution parameters.

### Data

We use the GTAP 6 data base which describes the world economy in 2001 and contains 87 regions and 57 sectors (Dimaranan 2006). We aggregate the regions and sector to focus on the water-using sectors in the Netherlands and its neighbors in the Rhine and Meuse river basin. The aggregated regions, sectors, and endowments used in this study are shown in Table 1.

**Table 1:** Aggregated regions, sectors, and endowments used in this study

Regions	Sectors
1. Netherlands	1. Wheat (genus: *Triticum*)
2. Belgium and Luxembourg	2. Cereal crops (family: Poaceae)
3. Germany	3. Vegetables and fruits^Ag^
4. France	4. Sugar beets^Ag^ (*Beta vulgaris*)
5. Rest of Europe	5. Other crops^Ag^
6. Rest of the World	6. Animal husbandry^Ag^
	7. Food products^Ind^
Endowments	8. Clothing and textiles^Ind^
1. Agricultural water volumes	9. Paper and pulp^Ind^
2. Dedicated agricultural water capital	10. Metal manufactures^Ind^
2. Irrigable land	11. Basic chemicals^Ind^
3. Rainfed land	12. Public water services^Ind^
4. Pasture land	13. Other manufacturing^Ind^
5. Labor	14. Other industry
6. Capital	14. Services
7. Industry water volumes	12. Transport
8. Dedicated industrial water capital	13. Capital goods

N.B. The superscripts on the sectors indicate which water endowment is used; Ag refers to the agricultural water endowment (Fig. 1) and Ind to the industry water endowment (Fig. 2). Sectors without a superscript do not use the water endowments. Wheat and cereal crops may, in general, be irrigated but are entirely rainfed in the Netherlands

To determine the share of the crop sectors’ use of irrigation water, irrigated land, and rainfed land, we used the procedure detailed in Calzadilla et al. (2011) and summarized as follows. First, the value of pasture land is simply the value of the original land endowment used by the animal husbandry sector. Then, for each crop sector, the remaining value of the original land endowment was split into irrigated land and rainfed land based on the volume of irrigated and rainfed production of the crop. Finally, the agricultural water composite was split off from the irrigated land endowment for each crop based on the ratio between rainfed and irrigated yields, where the value of the additional yield gain is attributed to irrigation water. We used data on irrigated and rainfed production and yields from the IMPACT model (IFPRI 2010) for all crop sectors and all regions except the Netherlands. For the Netherlands, we used data from a more detailed study of Dutch crop yields from Deltares (2013) because that seemed to be more accurate (for a detailed discussion, see Koopman et al. (2015)). To estimate the value of the agricultural water composite for the animal husbandry sector (which is split off from the value of pasture land), we used data on the volumes of water used for animal husbandry and also for irrigated crops. Then, we assumed that average value of a cubic meter of water in the animal husbandry sector is the same as in the crop sectors (see below for data sources on water volumes).

As far as we are aware, there are no studies which explicitly examine the value of water for manufacturing sectors in the Netherlands and Europe. However, there are a few studies on water use characteristics in manufacturing (see, for example, Reynaud (2003) for France and van der Zeijden et al. (2009) for the Netherlands). To determine the value of the water composite for the manufacturing sectors, we used a survey by Scharf et al. (2002) of Canadian manufacturers, which details expenditure on water extraction, treatment, recirculation, and discharge for several manufacturing sectors. We transferred these expenditures to our model regions by using the number of employees per manufacturing sector from Scharf et al. (2002) and Eurostat (Eurostat 2012) as a scaling factor. The value of the industry water composite for the Rest of the World (RoW) region (Table 1) was determined by imposing the same ratio of the value of the water composite to the value of capital as the rest of Europe region. Table 2 shows the total calculated expenditure on water abstraction, treatment, and discharge of water users in the manufacturing sectors in the Netherlands as well as the total output of each sector. For the value of the water composite in the public water services sector (not shown in Table 2), we relied on Teeples and Glyer (1987) who estimated a constant cost share of raw water of 18 %.

**Table 2:** Expenditures on water abstraction treatment and discharge for all manufacturing sectors

Sector	Food products	Clothing and textiles	Paper products	Metal manufactures	Basic chemicals	Other manufactures
Expenditure on water intake, treatment, and discharge (million USD)	40.4	5.6	221.9	112.2	44.0	30.3
Value of sector output (million USD)	42,791	4159	20,176	21,572	39,173	59,150
Share of water in total expenditure (%)	0.10	0.13	1.2	0.52	0.11	0.05

N.B. The value of total output (total expenditure) in US dollars (USD) of each sector is also displayed as well as the share of water in total expenditure. Source: Scharf et al. 2002 and own computations

For volumes of water used in the manufacturing and public water services sectors, we used data from the Eurostat website for all regions in the study with the exception of the RoW. We used the Eurostat website (Eurostat 2014) for irrigated water volumes as well, with the exception of the Netherlands where we used data from Hoogewoud et al. (2013). The irrigated volumes from Hoogwoud et al. differ substantially from the Dutch irrigation data from the Eurostat website, but we use Hoogwoud et al. because it is consistent with Deltares (2013) which we use for the value of Dutch crop production and the direct effects of climate change on agriculture (Sect. 3).^1^

For volumes of water used in the animal husbandry sector, we used the report by Ward and McKague (2007) on water consumed per head of various livestock types and then used the Eurostat website (Eurostat 2013) for the number of standardized livestock heads per region. The volumes of water used for the Rest of the World (RoW) region were estimated such that the RoW region had the same ratio of water to land, for the agricultural sectors, and water to capital, for the industrial sectors, as the Rest of Europe region.

In estimating the water volumes used by industry, we ignore water used for cooling, which takes place mostly in the energy sector, but also in certain types of manufacturing. Water used for cooling is a process which involves abstracting large volumes of surface water, but returning virtually the same quantity and quality, only slightly warmer. As water use for cooling involves very little consumptive use, it does not necessarily involve a trade-off between users. An exception is if the water temperature is already quite high, then warmed water could affect the ecosystem where it is discharged. Trade-offs between water for environmental and economic use and the resultant feedbacks of water quality on economic use are beyond the scope of this paper (see Brouwer et al. (2008), Dellink et al. (2011), and Zhu and van Ierland (2012) for studies that include water quality in assessments of water for economic use).

We were not able to obtain data on volumes of water used for individual crop and manufacturing sectors only on the total amount of water abstracted in manufacturing and separately for irrigation. We made the simplifying assumption that the average (shadow) value of a cubic meter of water is the same for all sectors within manufacturing and within irrigated crop categories. The value of the water use composite and the volumes of water used in each of these categories for the Netherlands are given in Table 3.

**Table 3:** The value of the water use composite and the volumes of water used in each of four use categories for the Netherlands in 2001

Water use category	Irrigated crops	Animal husbandry	Manufacturing	Public water services	Total
Volume abstracted (million m^3^)	271	7	121	1303	1702
Initial value of endowments (million USD)	88	2	454	280	824
Average value (USD m^−3^)	0.32	0.32	3.75	0.22	0.48

N.B. The bottom row displays the average value (or expenditure) per cubic meter of water used in the four use categories. Source: multiple sources (see text)

The volumes of water used and the associated value of the water endowments shown in Table 3 are for self-abstracted water only. All use of purified water purchased from third parties including all drinking water is incorporated into public water services sector. The Netherlands has a very extensive piped water network. The irrigated crops and animal husbandry sectors use quite a bit of drinking water. This may account for the rather low volumes of self-abstracted water used in the animal husbandry sector.

The average price per volume for drinking water deliveries charged to bulk water users by the Dutch public water utilities in 2001 is approximately 0.90 USD m^−3^ (Vewin 2002). Comparing this to the values given in Table 3, we see that the average value per cubic meter of self-abstracted water for agricultural users (0.32 USD m^−3^) is well below this price. However, the average value per cubic meter of self-abstracted water for the manufacturing sectors (3.75 USD m^−3^) is much higher than the drinking water price. This is due to the fact that most of the expenditure of manufacturing sectors on water use is on water treatment prior to discharge (Scharf et al. 2002).

In the Netherlands, there is no market for self-abstracted raw water; in fact, the government technically owns the water and allows firms to abstract it for their own use. Determining the exact value share of the water composite that should be attributed to the water volume endowment if the firms were to be granted property rights over the water that they use is beyond the scope of this paper. Therefore, to determine the value of the physical water volumes for each sector such that the markets clear in the benchmark equilibrium (or alternatively that the government has allocated water according to its shadow price), we use the procedure outlined in Appendix 2. The robustness of the results in the paper related to the assumptions made in Appendix 2 is given in Appendix 3.

## Climate change and water allocation scenarios

Climate change can have ambiguous effects on global markets. For example, countries around the Mediterranean might have a serious reduction in agricultural production from higher temperatures and reduced rainfall, but in contrast, warmer temperatures might create very favorable agricultural conditions in Greenland and other northern areas. Similarly, rising sea levels might threaten many world ports, but warmer temperatures might also open up new trade routes in the arctic, reducing transportation costs.

For a low-lying country such as the Netherlands, sea and river flooding is generally considered to be the most serious threat. Yet, one of the four climate change scenarios that were drafted by the Netherlands Royal Meteorological Institute (KNMI) predicts substantially warmer and dryer summers and a significant decrease in summer discharge by 2050 of two of the Netherlands’ major rivers, the Rhine and the Meuse (van den Hurk et al. 2006; te Linde 2007). The threat of water scarcity brings challenges to many aspects of Dutch society, from the supply of drinking water to production in agriculture and industry, the health of ecological systems, and the integrity of water infrastructure such as levees and dikes (Klijn et al. 2011). The starting point for our policy simulations is the Dutch climate change scenario W+. It is one of four scenarios deemed equally plausible for the Netherlands in 2050 (van den Hurk et al. 2006). The W+ scenario predicts a 2.8 °C increase in the average summer temperature and a 19 % decrease in the average summer precipitation. The summer discharge of the Rhine and the Meuse rivers, responsible for almost all of the water inflow into the Netherlands, drops by 35 and 20 %, respectively (te Linde 2007). We assume that these climate and river discharge conditions will extend across the entire international Rhine and Meuse river basins.

The hotter and drier W+ climate will impact the productive capacity of the economy in two particular ways: higher temperature and reduced precipitation negatively impact the productivity of land for agricultural use (Calzadilla et al. 2014), and reduced precipitation and river discharge lead to less water being available as an input for economic use.

To estimate the severity of the loss of land productivity and available water, we rely on the results of the Delta Program (Deltares 2013). That study used crop growth models to estimate the yield loss due to the W+ climate change scenario assuming no economic adaptation. The only adaptation possibilities were that farmers could irrigate their crops within the constraints of existing infrastructure and available water.

Following the procedure detailed in Koopman et al. (2015), we calculate the equivalent loss of water availability and land productivity within the GTAP production function (made suitably inflexible to mimic the adaptation restrictions of the crop growth models) that would result in the same production losses as in Deltares (2013).

The estimated losses in land productivity and total freshwater for economic use as a result of the W+ water scarcity scenario are shown in Table 4. It is the shocks in Table 4 which we impose on the model to simulate the water scarcity effects of climate change. Although the land productivity and irrigation water losses shown in Table 4 were calibrated from data in the Netherlands only, we make the simplifying assumption that the other regions in the Rhine and Meuse river basins experience the same change in climate and the same loss in land productivity.

**Table 4:** Percentage change in irrigated, rainfed, and pasture land productivity and volume of summer raw water from the W+ climate change scenario

	Irrigated land productivity	Rainfed land productivity	Pasture land productivity	Volume of summer raw water
Wheat	0	−9.5		
Cereal crops	0	−9.5		
Vegetables and fruits	−3.8	−23.4		
Sugar beets	−11.6	−19.6		
Other crops	−9.5	−20.2		
Animal husbandry			−3	
Water endowment				−11

Source: Deltares (2013) and own computations

In estimating the loss of freshwater for economic use, we are estimating the loss of available water, at the time and place that it is demanded, under current infrastructure conditions. The W+ scenario predicts increased water availability in the winter. We assume, however, that extra winter water cannot be used in the summer in excess of what is already being stored under the current infrastructure. The results from the Delta Program (Deltares 2013) suggest an 11 % loss in effectively available irrigation water in the summer (we assume that all irrigation occurs in the summer). We assume that the W+ scenario causes a proportional loss of water available for the other three water use categories in Table 3, implying that there will be an 11 % loss to the entire summer water supply available for economic use. We assume that the animal husbandry, public water services, and manufacturing sectors have a constant requirement for water use throughout the year, and so, the 3-month summer water requirements of these sectors is 25 % of their yearly water requirement, and the value of the summer water endowment is 25 % of the value of the water endowment in 2001 shown in Table 3. To ensure that only summer production is affected by the summer water reductions, we divided all water-using sectors into a summer fraction and a rest-of-the-year fraction (with the exception of the irrigated agricultural activities whose loss of yearly output was calibrated to a loss of summer irrigation water from Deltares 2013). Only the summer fraction of manufacturing, animal husbandry, and public water services is affected by water scarcity.

Given the climate impacts of the W+ climate change scenario on water resources and land productivity (Table 4), we simulate four separate policy scenarios of various water markets and evaluate their potential as an adaptation response to water scarcity. In all four scenarios, the productivity shocks from Table 4 are imposed on agriculture and an 11 % loss of summer water is imposed in equal measure on all water-using sectors.

### No water market (no water market)

In the no-water-market scenario, there is no possibility for exchanging water through a water market between sectors. That is the water volume endowment is made immobile.

### Two water markets (two markets)

In the two-water-market scenario, two distinct water markets are specified. In this case, the agricultural water market supplies the agricultural sectors (including animal husbandry) and the industry water market supplies the manufacturing and public water services sectors, but without possibilities of exchanging water between industry and agriculture. The water volume endowment is mobile, but a distinction is made between the water volume endowment that serves the industry water market and the water volume endowment that serves the agriculture water market.

### Single water market (single market)

In the single-water-market scenario, there is one water market that supplies all sectors. This is the most flexible option, where water can also be exchanged between sectors through the creation of a single market. The water volume endowment is mobile, and no distinction is made between the water volume endowments that serve the industrial or agricultural sectors.

### Single water market without public water services (single market without public water services)

Perhaps for political or equity reasons, policy makers might find additional price increases for public water services unacceptable. Therefore, in this policy scenario, there is a single market for water-using sectors; however, the public water services sector does not participate. The public water services sector receives an 11 % loss of summer water availability while the remaining summer water-using sectors collectively receive an 11 % loss of summer water and reallocate the remaining water among themselves within a single market. By removing it from the water market, public water services in essence receive a subsidy such that the shadow price of water that it faces is lower than the market price of water.

In all policy scenarios, the other regions in the Rhine-Meuse river basin are assumed to have the water allocation policy described in the no-water-market scenario; i.e., water cannot be exchanged between sectors.

## Results

In the no-water-market scenario, the supply of water falls by an equal percentage in all water-using sectors shown in Table 5. In response to the drop in water supply, the shadow price of water rises by far the highest in the manufacturing sectors followed by the agricultural sectors and less in the public water services. This is shown in Table 6 which displays the percent change in the (shadow) price of water for each scenario relative to the percent change in the price of water in the single-market scenario. The underlying reason for the difference in the change in shadow price of water in response to the same percentage reduction in water quantity can be found in Table 3 of Sect. 2.3. From Table 3, we see that the average value of a cubic meter of water used in public water services is lower than in agriculture and much lower compared with manufacturing. Thus, the productivity of an additional unit of water under the water scarcity shock is much higher in manufacturing than in agriculture or public water services.

**Table 5:** Percentage change in quantity of water use under the W+ climate change water scarcity scenario under four different water market alternatives

	No water market	Two markets	Single market	Single market without public water services
Vegetables and fruits	−11	−10	−7	−11
Sugar beets	−11	−7	−5	−8
Other crops	−11	−12	−9	−13
S. Animals	−11	−17	−7	−20
S. Food products	−11	0	−1	−1
S. Clothing and textiles	−11	0	−1	−1
S. Paper and pulp	−11	0	−1	−1
S. Metal manufactures	−11	0	−1	−1
S. Basic chemicals	−11	0	−1	−1
S. Public water services	−11	−12	−15	−11
S. Other manufacturing	−11	0	−1	−1

N.B. Sectors with an S. before the name indicate that this is the summer fraction of the sector

**Table 6:** The ratio of the percentage change in the price of water over the percentage change in price of water in the single-market scenario

	No water market	Two markets	Single market	Single market without public water services
Vegetables and fruits	2.2	2.0	1.0	2.4
Sugar beets	3.1	2.0	1.0	2.4
Other crops	1.8	2.0	1.0	2.4
S. Animals	1.3	2.0	1.0	2.4
S. Food products	16.4	0.8	1.0	2.4
S. Clothing and textiles	16.5	0.8	1.0	2.4
S. Paper and pulp	16.4	0.8	1.0	2.4
S. Metal manufactures	16.4	0.8	1.0	2.4
S. Basic chemicals	16.5	0.8	1.0	2.4
S. Public water services	0.8	0.8	1.0	0.8
S. Other manufacturing	16.5	0.8	1.0	2.4

N.B. Sectors with an S. before the name indicate that this is the summer fraction of the sector

The larger economy-wide changes for the Netherlands resulting from the climate shock and the four water market configurations are shown in Table 7. The disaggregated output and price changes per sector can be found in Appendix 1. In the no-water-market scenario, GDP output has the largest loss of all the various water market configurations. Agriculture loses 2.19 % of its output; by far, the largest of all the water use categories, public water services lose 0.54 % of its output, comparatively little, while manufacturing actually gains overall from the water-scarce climate. This is not surprising as agriculture endures a loss of land productivity in addition to the loss of available water. Further, the various land and water endowments in agriculture account for approximately 16 % of the total value added in production for the irrigated activities and 60 % of the rainfed activities (wheat and cereal crops). In contrast, the percentage of the industrial water composite in total value added in the manufacturing sectors ranges between 4 % (paper and pulp) and 0.3 % (other manufacturing). The fraction for public water services is higher (30 %); however, the final demand for public water services is highly inelastic (Arbues et al. 2003) which results in higher prices for public water services when supply falls. The increased supply price for public water services allows the sector to increase its use of water substitutes (labor and capital) which in turn allows the sector to keep the reduction in supply relatively modest.

**Table 7:** Economy-wide results for the Netherlands under the W+ climate change water scarcity scenario

	No water market	Two markets	Single market	Single market without public water services
Percent change in output				
GDP	−0.024	−0.021	−0.021	−0.021
Agriculture	−2.20	−2.25	−2.07	−2.33
Manufacturing	0.04	0.06	0.05	0.07
Public water services	−0.54	−0.59	−0.72	−0.54
Percent change in price				
Capital	−0.17	−0.15	−0.15	−0.16
Labor	−0.16	−0.15	−0.14	−0.15

The large drop in the output of agriculture reduces demand for labor and capital services which in turn reduces their market prices (wages). The reduced price of labor and capital services more than makes up for the loss of water for manufacturing as a whole. Therefore, manufacturing faces more favorable economic conditions in the water-scarce climate.

In the two-market scenario, the water supply is reduced by the same amount as in the no water market; however, two market mechanisms now exist, respectively, between the agricultural sectors and the industrial water users. As a result, the price of physical water is equalized separately within the agricultural and the industrial water-using sectors. From Table 5, we see that water moves from the public water services sector to the manufacturing sectors which have essentially no loss of water with respect to the benchmark equilibrium. Within the agricultural market, water moves from the animals sector and other crops to the vegetables and fruits and sugar beets sectors. The reallocation of water in the two-market scenario follows the difference in water shadow price from the no-water-market scenario shown in Table 6. Table 6 also shows that the percent change in water price is substantially higher in the agricultural market than in the industry water market which gives further insight into how water will move in the single-market scenario.

The aggregated economy-wide results from Table 7 show that in the two-market scenario, the GDP output improves compared with the no-water-market scenario, and access to more water allows the manufacturing sectors to further increase their output. More productive allocation of water also increases the demand for labor and capital which increases their price compared with the no-water-market scenario. This results in higher costs for agricultural inputs which further depress output. Sharing a water market with manufacturing causes public water services to pay a slightly higher price for its water in addition to a higher price for labor and capital than in the no-water-market scenario which also causes a reduction in output.

In the single-market scenario, all water-using sectors participate in a unified market resulting in a single price for water across all uses. In this scenario, water moves from public water services into agriculture as well as into manufacturing. The agricultural sectors use more water than in the two-market scenario while manufacturing sectors use slightly less, as the water demand from agriculture drives up the price that the manufacturing sectors face for water volumes. Public water services use even less water than in the two-market scenario.

The single-market scenario is the best outcome for agriculture, as agriculture has the highest output in the single-market scenario compared with all other scenarios. Manufacturing does slightly worse than in the two-market scenario, and public water services have the largest loss in the single-market scenario compared with all other scenarios, as it faces higher prices for water, labor, and capital than in the other market scenarios.

Finally, we examine the scenario where there is a single market for water, but public water services do not participate and so only suffer the direct loss of 11 % of its raw water supply. From Table 5, we see that as the water from public water services is not available, water moves from the agricultural activities to the manufacturing sectors. On the whole, agriculture has less water available to it than in the no-water-market scenario, but the remaining water is more productively allocated across the agricultural activities. The more efficient allocation of water within agriculture is not enough to offset the additional loss of water. In terms of total output, this is the worst scenario for agriculture. Manufacturing gains the most from this scenario, as the depressed price of labor and capital more than compensate for the higher price for water compared with the single-market and two-market scenarios.

Table 8 shows the impact of the W+ climate change scenario on the upstream river basin regions Belgium-Luxembourg, Germany, and France. All these countries suffer the same climate shock as the Netherlands, and all have the no-water-market policy scenario. All countries have the same or slightly higher GDP output loss as compared to the Netherlands, and all countries have a larger percent loss of agricultural output. Compared to the other countries in the river basin, Dutch agriculture is relatively protected from the water-scarce climate shock because a larger share of Dutch agriculture is irrigated. Implementation of various water market constructions in the Netherlands has little effect on the upstream neighbors with the exception of the single-market scenario. This scenario reduces the loss of Dutch agriculture to a significant degree (compared with the other three market scenarios) that it has an impact on agricultural markets of its large trading partners in the river basin.

**Table 8:** Percent output loss of GDP and agriculture for the upstream countries under the W+ climate change water scarcity scenario

	Single market			All other market scenarios		
	Belgium-Luxembourg	Germany	France	Belgium-Luxembourg	Germany	France
GDP	−0.023	−0.032	−0.046	−0.023	−0.032	−0.046
Agriculture	−3.15	−3.52	−2.94	−3.10	−3.48	−2.94

The upstream countries all have the no-water-market policy scenario. The single-market and all other market scenarios refer to the Netherlands policy scenario

## Discussion and conclusions

In the Netherlands, most economic analysis in the water area is focused on water supply measures. For example, van Beek et al. (2008) carried out a cost-benefit analysis for several large-scale water infrastructure projects in the Netherlands with the aim of increasing the reliability of irrigation water supplies. There is less focus on the demand side and demand management options. The most important demand side tool that is available is the priority list for water use in times of scarcity that was instituted by the Dutch government after the severe drought in 1976 and that was updated after the very dry summer of 2003 (Arcadis 2012). This priority list recognizes that during times of scarcity, water should be allocated first to higher-value economic and environmental activities but gives little detail or prescription on its implementation.

To explore the impact of water scarcity from climate change on the Dutch economy and the adaptation capabilities of water markets, we have extended the GTAP-W model by including explicit water use in the manufacturing, public water service, and animal husbandry sectors in addition to the irrigated crop sectors. We have further extended the GTAP-W model by linking the value of the water activity in production to the volume of water used and introducing a market that allows for trading of raw water across different uses while enforcing the physical water balance.

The results show that almost all of the losses in production to the Dutch water-using economic sectors are in agriculture which loses 2.19 % of its output in response to the climate conditions as predicted in the W+ scenario with warmer and dryer summers in the Northwest of Europe. GDP output falls by approximately 0.024 %. The manufacturing sectors and other non-agricultural sectors make use of depressed wages and capital rentals to mitigate their losses or even increase their output. Instituting any of the three water markets considered in this paper reduces the GDP loss slightly to 0.021 %.

Even though instituting a water market increases overall output, any particular instance of the three-water-market possibilities considered will have winners and losers from the perspective of sector output. The particular winners and losers can be seen by examining the shadow price of water for each sector in the no-water-market scenario (Table 6). If a sector’s water shadow price is lower than the shadow prices of the other possible participants in a market or lower than the eventual market price of water in any instance of market, then the sector will loose from creation of a market. On the other hand, any sector with a higher shadow price for water than the eventual market price will gain. Put another way, a sector with a water shadow price in the no-market scenario that is lower (higher) than the market price for water that would exist in the instance of a particular water market is essentially receiving a subsidy (tax). Creating that water market would remove the subsidy or tax. Examining the water shadow prices in the no-market scenario and the results from the three-water-market scenarios, we see that the manufacturing sectors always gain from each of the three market possibilities. Public water services always lose and agriculture gains only if it is in a market with public water services.

Instituting a water market can also have an impact on the water-using sectors of the upstream countries which share the river basin via trade linkages (there are no physical water linkages between countries in this study). Although these effects are small, it could still be an important political consideration in the construction of a national water market because in times of water scarcity, the upstream countries could conceivably take more than their expected or agreed upon share of water.

The methodology used in this study has as usual some limitations. For instance, we assume the absence of transaction costs in any water exchange and, furthermore, that sufficient infrastructure exists such that additional water can be abstracted at the point of use in any exchange of water abstraction permits. This assumption would overestimate the benefits of water markets. On the other hand, we assume a common shadow price for water in the benchmark equilibrium. If water abstraction is charged by volume, it is much more likely that agriculture will be charged water at a lower price than industry or public water services; if the baseline already contains this imbalance, then a water market would provide additional benefits not captured by this study.

As we do not know the producer’s shadow value of water volumes, we used as proxies the money spent on machinery necessary for water’s use in production for industry and the additional value of irrigation for agriculture. We then separated the value of machinery from the value of water (Appendix 2) in such a way that the exact value attributed to water had little impact on the results (Appendix 3 for results on the robustness of this method). This was, however, an imperfect process, and additional information on the shadow value of physical water in production would be a great benefit to this study.

An interesting extension to this model would be to distinguish water by river basin source and to restrict the trade in water permits to users in the same river basin or to explicitly model the transportation costs of water. This study also ignores water used for cooling purposes as well as the demand for water by nature for a healthy ecosystem. Demand for water by nature in particular is likely to increase in a hotter and drier climate. Integrating the demand for water by nature into an analysis of water demands for the production of economic goods would also be a fruitful area for further research. We also do not include climate change impacts on countries outside of the Rhine-Meuse river basin. Including these additional climate impacts (both positive and negative) on the RoW would certainly have additional influence on the competitive position of Dutch products on world markets as well as the prices for foreign goods faced by Dutch consumers.

This study assumes perfect mobility of labor and capital; this assumption might overestimate the ability of the manufacturing sectors to capitalize on the depressed wages and returns to capital that result from a contracting agricultural sector and might thus underestimate the costs of the described climate change impacts.

Finally, we noticed a lack of research and data on industrial use of freshwater. Given the growing international concerns about emerging water scarcity because of climate change, there seems to be much value in further research in this area. Our model would certainly benefit from more robust data.

## Appendix 1: Quantity and price changes for all Dutch economic sectors for all water market scenarios

**Table 9:** The sector price and output change (in percent change) for each water market scenario for the Dutch economy.

	No water market		Two markets		Single market		Single market without public water services	
	Quantity	Price	Quantity	Price	Quantity	Price	Quantity	Price
Wheat (genus: *Triticum*)	−5.7	3.1	−5.7	3.1	−5.8	3.1	−5.6	3.0
Cereal crops (family: Poaceae)	−3.3	3.9	−3.3	3.9	−3.3	3.9	−3.2	3.8
Vegetables and fruits	−3.7	2.4	−3.3	2.2	−2.8	2.0	−3.5	2.3
Sugar beets (*Beta vulgaris*)	−0.5	3.9	−0.5	3.9	−0.6	3.9	−0.5	3.8
Other crops	−4.4	2.3	−4.8	2.4	−4.5	2.3	−5.0	2.4
S. Animals	−0.4	0.6	−0.4	0.6	−0.4	0.6	−0.4	0.6
Os. Animals	−0.5	0.5	−0.5	0.6	−0.5	0.6	−0.5	0.5
S. Food products	−0.4	0.2	−0.3	0.2	−0.4	0.2	−0.3	0.2
Os. Food products	−0.4	0.2	−0.4	0.2	−0.4	0.2	−0.4	0.2
S. Clothing and textiles	0.1	0	0.3	−0.1	0.2	−0.1	0.3	−0.1
Os. Clothing and textiles	0.3	−0.1	0.3	−0.1	0.3	−0.1	0.3	−0.1
S. Paper and pulp	−0.2	0.1	0.2	−0.1	0.2	−0.1	0.2	−0.1
Os. Paper and pulp	0.1	−0.1	0.1	−0.1	0.1	−0.1	0.1	−0.1
S. Metal manufactures	0.0	0	0.4	−0.1	0.4	−0.1	0.4	−0.1
Os. Metal manufactures	0.3	−0.1	0.3	−0.1	0.3	−0.1	0.3	−0.1
S. Basic chemicals	0.1	0	0.2	−0.1	0.1	−0.1	0.2	−0.1
Os. Basic chemicals	0.1	−0.1	0.1	−0.1	0.1	−0.1	0.1	−0.1
S. Public water services	−1.5	12.3	−1.7	13.6	−2.1	17.4	−1.5	12.2
Os. Public water services	−0.2	0.1	−0.2	0.1	−0.3	0.2	−0.2	0.1
S. Other manufacturing	0.2	−0.1	0.2	−0.1	0.2	−0.1	0.2	−0.1
Os. Other manufacturing	0.3	−0.1	0.2	−0.1	0.2	−0.1	0.3	−0.1
Other industry	−0.1	−0.1	−0.1	−0.1	−0.1	−0.1	−0.1	−0.1
Services	0	−0.1	0	−0.1	0	−0.1	0	−0.1
Transport	0.1	−0.1	0.1	−0.1	0.1	−0.1	0.1	−0.1
Capital goods	−0.1	−0.1	−0.1	−0.1	−0.1	−0.1	−0.1	−0.1

Where sectors have been separated into summer portion and other seasonal portion, S. sector denotes the summer portion and Os. sector denotes the other seasonal portion of the sector

## Appendix 2

The procedure to determine the value of physical water volumes for each sector such that water markets clear (or alternatively that water has been allocated according to its shadow price) in the benchmark equilibrium is as follows. We identify the water use category which has the water composite with the lowest average value per cubic meter of water. For the Netherlands and Germany, this is public water services, and for France and Belgium, this is irrigated crops. We then use this category as a marker and split the value of the water composite arbitrarily at 75 % attributed to the water volumes and 25 % attributed to dedicated water capital endowments. Then, with a specific value attached to the water volume endowment of public water services, we divide by the volumes of water used to that we now have an average value of water per cubic meter used. Finally, we assume that the average value of water is the same across all uses in the benchmark equilibrium, and for all other water use categories, we multiply this average value per cubic meter by the number of cubic meters used in production. We then have a value attributed to the total water volume endowment for every water use category and use the ratio of this value over the total value of the water use composite for each water use category to find the value share attributed to water volumes for each sector.

The initial split for the marker water use category is admittedly arbitrary; however, the no-substitution condition between the water volumes and the dedicated water capital endowments imposed in the model insures that percentage reduction in the water volume endowment results in the same percentage reduction in the water composite one step higher in the nest regardless of the value share attributed to the water volume endowment. While the magnitude of some of the results does change slightly according to the percentage of value of the water composite attributed to water volumes, these changes are small with the exception of the price variable of water volumes. The magnitude of the percent change in the price of water is very dependent on the share of the value attributed to it, and so, we have chosen to present the change in price of water volumes as a relative change using the price change in a particular scenario as a numeraire. The trends of the results are independent of the value share of water volumes. The results presented in Sect. 4 were achieved by attributing 75 % of the value of the water composite to the water volume endowment for the marker category. Appendix 3 presents the same results when the attribution is 50 and 95 %, respectively. For the Netherlands, using public water services as the marker and using the value and volume data from Table 3, attributing 75 % of the value of the water composite to the water volume endowment results in attributing 50 % of the value of the water composite to the water volume endowment for irrigated crops and animal husbandry and 4 % for the manufacturing sectors.

## Appendix 3

The following gives an indication of the robustness of the results with respect to the share of value of the water composite attributed to water volumes (see Appendix 2). This is done by presenting the results from Sect. 4, with a 50 % value share attributed to public water services and a 95 per value share attributed to public water services.

**Table 10:** Percent change in quantity of water use under the W+ climate change water scarcity scenario

	No water market		Two markets		Single market		Single market without public water services	
	95 % split	50 % split	95 % split	50 % split	95 % split	50 % split	95 % split	50 % split
Vegetables and fruits	−11	−11	−10	−10	−6	−7	−11	−11
Sugar beets	−11	−11	−7	−8	−5	−5	−8	−8
Other crops	−11	−11	−12	−12	−9	−9	−13	−13
S. Animals	−11	−11	−17	−17	−7	−7	−20	−20
S. Food products	−11	−11	−0	−1	−0	−1	−1	−1
S. Clothing and textiles	−11	−11	−0	−1	−0	−1	−1	−1
S. Paper and pulp	−11	−11	−0	−1	−0	−1	−1	−1
S. Metal manufactures	−11	−11	−0	−1	−0	−1	−1	−1
S. Basic chemicals	−11	−11	−0	−1	−0	−1	−1	−1
S. Public water services	−11	−11	−12	−12	−14	−15	−11	−11
S. Other manufacturing	−11	−11	−0	−1	−0	−1	−1	−1

N.B. Sectors with an S. before the name indicate that this is the summer fraction of the sector. The results are displayed for the case where 95 and 50 % of the value of the water composite are attributed to water volumes

**Table 11:** For each of the four water market scenarios, Table 6 shows the ratio of the percent change in price of water over the percent change in price of water in the single-market scenario

	No water market		Two markets		Single market		Single market without public water services	
	95 % split	50 % split	95 % split	50 % split	95 % split	50 % split	95 % split	50 % split
Vegetables and fruits	2.4	2.0	2.2	1.8	1.0	1.0	2.7	2.2
Sugar beets	3.5	2.8	2.2	1.8	1.0	1.0	2.7	2.2
Other crops	2.0	1.7	2.2	1.8	1.0	1.0	2.7	2.2
S. Animals	1.4	1.3	2.2	1.8	1.0	1.0	2.7	2.2
S. Food products	19.3	13.8	0.8	0.8	1.0	1.0	2.7	2.2
S. Clothing and textiles	19.5	13.9	0.8	0.8	1.0	1.0	2.7	2.2
S. Paper and pulp	19.3	13.8	0.8	0.8	1.0	1.0	2.7	2.2
S. Metal manufactures	19.4	13.8	0.8	0.8	1.0	1.0	2.7	2.2
S. Basic chemicals	19.5	13.9	0.8	0.8	1.0	1.0	2.7	2.2
S. Public water services	0.7	0.8	0.8	0.8	1.0	1.0	0.7	0.8
S. Other manufacturing	19.5	13.9	0.8	0.8	1.0	1.0	2.7	2.2

N.B. Sectors with an S. before the name indicate that this is the summer fraction of the sector. The results are displayed for the case where 95 and 50 % of the value of the water composite are attributed to water volumes

**Table 12:** Economy-wide results for the Netherlands under the W+ climate change water scarcity scenario

	No water market		Two markets		Single market		Single market without public water services	
	95 % split	50 % split	95 % split	50 % split	95 % split	50 % split	95 % split	50 % split
Percent change in output								
GDP	−0.024	−0.024	−0.021	−0.021	−0.021	−0.021	−0.021	−0.021
Agriculture	−2.20	−2.20	−2.25	−2.25	−2.07	−2.07	−2.33	−2.33
Manufacturing	0.04	0.04	0.06	0.06	0.05	0.05	0.07	0.07
Public water services	−0.54	−0.54	−0.59	−0.59	−0.72	−0.73	−0.54	−0.54
Percent change in price								
Capital	−0.17	−0.17	−0.15	−0.15	−0.15	−0.15	−0.16	−0.16
Labor	−0.16	−0.16	−0.15	−0.15	−0.14	−0.14	−0.15	−0.15

The results are displayed for the case where 95 and 50 % of the value of the water composite are attributed to water volumes

## Footnotes

1 See Hoogewoud et al. (2013) for a discussion on the cause of the differences between its results and the data reported by Eurostat.
